# Commentary: Blood Flow Restriction Exercise: Considerations of Methodology, Application, and Safety

**DOI:** 10.3389/fphys.2020.599592

**Published:** 2020-11-19

**Authors:** Marty D. Spranger

**Affiliations:** Department of Physiology, Michigan State University, East Lansing, MI, United States

**Keywords:** blood flow restriction (BFR), exercise pressor reflex, cardiovascular disease, muscle metaboreflex activation, occlusion training

Blood flow restriction (BFR) training is a bourgeoning exercise modality which promotes increases in skeletal muscle mass and strength during resistance exercise (Sato, 2005). In practice, prior to a bout of exercise, a pneumatic cuff (or tourniquet) is inflated around the limb proximal to the muscle to be trained thereby restricting its blood flow. During exercise with BFR, the ischemic working muscle inordinately accumulates metabolites (e.g., proton and lactate) which promote muscle strength and hypertrophy (Slysz et al., 2016). The metabolic environment of the muscle interstitium during non-restricted, high-intensity resistance exercise can be simulated by performing blood flow-restricted low-intensity resistance exercise (Kim et al., 2017). BFR resistance exercise (BFR-RE) is employed by bodybuilders, athletes, and is also intriguing for individuals with limited or incapacity of performing high-intensity resistance exercise: elderly (Centner et al., 2019) and clinical populations such as orthopedic (Hughes et al., 2017) and cardiac rehabilitation (Madarame et al., 2013; Kambic et al., 2019) patients.

Spranger et al. (2015) offered caution to a hasty, wholesale implementation of BFR training from gym to clinic. The exercise pressor reflex (EPR—muscle metaboreflex and muscle mechanoreflex) was the primary basis of this concern. As previously stated, metabolites accumulate in the muscle interstitium during exercise. The muscle interstitium contains chemically sensitive receptors within group IV afferent nerve fibers which respond to these metabolites, reflexively engaging the metaboreflex. In turn, the metaboreflex increases sympathetic outflow leading to marked increases in hemodynamics such as heart rate and arterial blood pressure (Boushel, 2010). Ischemic exercise, such as BFR-RE, inordinately accumulates metabolites within the muscle interstitium (Takarada et al., 2000; Takada et al., 2012), supporting the contention that BFR-RE leads to exaggerated metaboreflex activation.

Several studies in young, healthy individuals have shown that BFR-RE exacerbates cardiovascular responses. Downs et al. (2014) reported that BFR-RE raised systolic blood pressure (SBP) (156 ± 7 mmHg) by ~29 mmHg vs. exercise without BFR (127 ± 4 mmHg). Prodel et al. (2016) reported that BFR-RE raised SBP (191 ± 8 mmHg) by ~38 mmHg vs. exercise without BFR (153 ± 7 mmHg). Thomas et al. (2018) reported that BFR-RE increased mean arterial pressure (MAP) by ~20 mmHg vs, exercise without BFR. Importantly, they further showed that low-intensity, BFR-RE increased MAP (124.2 ± 2.3 mmHg) by ~10 mmHg vs. high-intensity, exercise without BFR (113.9 ± 2.5 mmHg). They concluded that practitioners should use caution if prescribing this BFR exercise modality within a vascular compromised population. Recently, Hori et al. (2020) reported that BFR-RE exaggerated SBP from rest to exercise (52.4 ± 6.9 mmHg) vs. exercise in the absence of BFR (27.0 ± 3.1 mmHg). Moreover, they provided the first evidence that the BFR-RE-induced exaggeration in SBP can be explained by the SBP response to post-exercise muscle ischemia, which elicits the metaboreflex (Crisafulli et al., 2018). These data, and other (Domingos and Polito, 2018; Scott et al., 2018; Cristina-Oliveira et al., 2020), demonstrate that BFR-RE can exacerbate metaboreflex activation leading to abnormal increases in hemodynamic responses during exercise.

While caution is warranted even in healthy individuals, the primary concern of Spranger et al. (2015) specifically pertained to patients with cardiac and vascular diseases, as metaboreflex hemodynamics are exaggerated in subjects with heart failure, hypertension, and peripheral artery disease during exercise. Therefore, caution is heightened when considering a BFR-RE regimen for these clinical populations (Spranger et al., 2015). In response to Spranger et al. (2015), Jessee et al. (2016) acknowledged this concern and proposed that making the arterial occlusion pressure relative to the cuff and individual may help reduce the risk of any adverse cardiovascular events associated with the EPR. Pope et al. (2013) furthered this sentiment, not only advocating that caution should be exercised for individuals with any form of cardiovascular disease, but also suggesting that BFR protocols for these individuals should employ reduced external cuff pressure, time under pressure, and physical exertion.

A few studies have employed BFR-RE in populations with cardiovascular disease with generally favorable conclusions. However, these conclusions were drawn despite the fact that either hemodynamics were not assessed in the study (Madarame et al., 2013; Groennebaek et al., 2019) or they were only assessed before and after, but not during the exercise (Barili et al., 2018; Kambič et al., 2020). For example, Kambič et al. (2020) assessed the safety of BFR-RE in patients with stable coronary artery disease. While they did not measure hemodynamics in these patients during exercise, they nonetheless concluded that BFR-RE exhibited favorable hemodynamic responses in patients with CAD, and therefore was proven to be safe. When considering the safety of BFR-RE for individuals with cardiovascular disease, it is paramount to understand what hemodynamic changes are occurring in the individual during the exercise. It is this ischemic maneuver, BFR, coupled with the resistance exercise, which leads to the increased accumulation of metabolites in the muscle interstitium which reflexively engage the metaboreflex leading to exaggerated increases in hemodynamics. Indeed, Pinto and Polito (2016) measured hemodynamics in hypertensive women with and without BFR. They reported that BFR-RE exaggerated the rise in SBP from rest to exercise (52.4 ± 6.9 mmHg) vs. exercise without BFR (27.0 ± 3.1 mmHg).

Patterson et al. (2019), proffers a prescription for BFR training for practitioners. Importantly, this review addresses several potential safety concerns to consider when coupling BFR to exercise. However, I am very concerned that Patterson et al. (2019) fail to mention and acknowledge the EPR, and even more specifically the metaboreflex, when addressing potential safety concerns regarding cardiovascular responses to BFR-RE. The studies provided in this commentary, and those thoroughly outlined by Cristina-Oliveira et al. (2020), clearly demonstrate that BFR-RE can markedly increase hemodynamic parameters, likely owing to exaggerated metaboreflex activity. This concern is amplified when considering the prescription of BFR-RE to individuals with cardiovascular disease.

Patterson et al. (2019) intend for their review to be a research-informed guide to BFR training for practitioners. I applaud the authors for their efforts and support Patterson et al. (2019) as “the guide to BFR exercise” moving forward. However, I contend that this “guide” is deficient in important safety information regarding BFR exercise and its interaction with the EPR. This is essential and vital knowledge for BFR practitioners, and thus, as in Vanwye et al. (2017), must be provided in the consequential review by Patterson et al. (2019).

## Author Contributions

The author confirms being the sole contributor of this work and has approved it for publication.

## Conflict of Interest

The author declares that the research was conducted in the absence of any commercial or financial relationships that could be construed as a potential conflict of interest.
